# CYP46A1 activation by low-dose efavirenz enhances brain cholesterol metabolism in subjects with early Alzheimer’s disease

**DOI:** 10.1186/s13195-022-01151-z

**Published:** 2022-12-29

**Authors:** Alan J. Lerner, Steven E. Arnold, Erin Maxfield, Aaron Koenig, Maria E. Toth, Brooke Fortin, Natalia Mast, Bianca A. Trombetta, John Denker, Andrew A. Pieper, Curtis Tatsuoka, Sangeetha Raghupathy, Irina A. Pikuleva

**Affiliations:** 1 Brain Health and Memory Center, Neurological Institute, University Hospitals Cleveland Medical Center, Cleveland, OH 44122 USA; 2 Department of Neurology, Case Western Reserve University, Cleveland, OH 44106 USA; 3 Alzheimer’s Clinical and Translational Research Unit, Massachusetts General Hospital, Charlestown, MA 02129 USA; 4 Department of Ophthalmology and Visual Sciences, Case Western Reserve University, Cleveland, OH 44106 USA; 5 Harrington Discovery Institute, University Hospitals Cleveland Medical Center, Cleveland, OH 44106 USA; 6 Department of Psychiatry, Case Western Reserve University, Cleveland, OH 44106 USA; 7 Geriatric Psychiatry, GRECC, Louis Stokes Cleveland VA Medical Center, Cleveland, OH 44106 USA; 8 Institute for Transformative Molecular Medicine, School of Medicine, Case Western Reserve University, Cleveland, OH 44106 USA; 9 Department of Population and Quantitative Health Sciences, Case Western Reserve University, Cleveland, OH 44106 USA

**Keywords:** CYP46A1, Efavirenz, Alzheimer’s disease, 24-Hydroxycholesterol, Stable isotope labeling kinetics

## Abstract

**Background:**

Efavirenz is an anti-HIV drug, and cytochrome P450 46A1 (CYP46A1) is a CNS-specific enzyme that metabolizes cholesterol to 24-hydroxycholesterol (24HC). We have previously shown that allosteric CYP46A1 activation by low-dose efavirenz in a transgenic mouse model of Alzheimer’s disease (AD) enhanced both cholesterol elimination and turnover in the brain and improved animal performance in memory tests. Here, we sought to determine whether CYP46A1 could be similarly activated by a low-dose efavirenz in human subjects.

**Methods:**

This pilot study enrolled 5 subjects with early AD. Participants were randomized to placebo (*n* = 1) or two daily efavirenz doses (50 mg and 200 mg, *n* = 2 for each) for 20 weeks and evaluated for safety and CYP46A1 target engagement (plasma 24HC levels). A longitudinal mixed model was used to ascertain the statistical significance of target engagement*.* We also measured 24HC in CSF and conducted a unique stable isotope labeling kinetics (SILK) study with deuterated water to directly measure CYP46A1 activity changes in the brain.

**Results:**

In subjects receiving efavirenz, there was a statistically significant within-group increase (*P* ≤ 0.001) in the levels of plasma 24HC from baseline. The levels of 24HC in the CSF of subjects on the 200-mg dose of efavirenz were also increased. Target engagement was further supported by the labeling kinetics of 24HC by deuterated water in the SILK study. There were no serious adverse effects in any subjects.

**Conclusions:**

Our findings suggest efavirenz target engagement in human subjects with early AD. This supports the pursuit of a larger trial for further determination and confirmation of the efavirenz dose that exerts maximal enzyme activation, as well as evaluation of this drug’s effects on AD biomarkers and clinical symptomatology.

**Trial registration:**

ClinicalTrials.gov, NCT03706885.

**Supplementary Information:**

The online version contains supplementary material available at 10.1186/s13195-022-01151-z.

## Background

Alzheimer’s disease (AD) is the most common cause of dementia [1], and it is well recognized that metabolic factors are major drivers of the disease. The involvement of cholesterol in AD pathogenesis is exemplified by APOE, the major cholesterol transport protein in the CNS, and by the high prevalence of the APOE ε4 allele in patients with late-onset AD [2]. In addition, lipid raft cholesterol modulates the processing of amyloid precursor protein into amyloid β (Aβ) peptides, a hallmark of AD [3–5], and multiple studies support the importance of cholesterol intake and serum cholesterol levels in AD [6, 7]. Herein, we report on the novel use of low-dose efavirenz (EFV), a non-nucleoside reverse transcriptase inhibitor given in higher doses to treat human immunodeficiency virus (HIV) infection. We suggest that low-dose EFV enhances cholesterol elimination from the CNS in subjects with early AD.

The human brain, which contains approximately 25% of the body’s total unesterified cholesterol [8], is separated from the systemic circulation by the blood–brain barrier, which is impermeable to cholesterol. Hence, brain cholesterol is synthesized locally, with elimination mainly (75%) an enzymatic process via conversion to 24-hydroxycholesterol (24HC) by cytochrome P450 46A1 (CYP46A1) [9–11]. Under normal conditions, human CYP46A1 is a CNS-specific neuronal enzyme [12, 13] controlling not only the major pathway of the brain cholesterol elimination [11] but also brain cholesterol turnover with tight coupling between cholesterol 24-hydoxylation and cholesterol biosynthesis to maintain homeostatic levels of brain cholesterol [14]. In AD, however, there is ectopic CYP46A1 expression in astrocytes and decreased expression in neurons [12, 13]. Nevertheless, CYP46A1 still controls brain cholesterol turnover in this disease [15, 16].

Once 24HC is formed, it rapidly diffuses out of the brain and reaches either the systemic circulation (99%) or the cerebrospinal fluid (CSF, 1%), with subsequent delivery to the liver for further biotransformation [17, 18]. 24HC in the systemic circulation serves as a marker for brain activity of CYP46A1 [19], although its plasma levels are affected by liver function (or the ratio between the brain weight and liver volume), body surface area, disease state, inflammation, dysfunction of the blood–brain barrier, and cholesterol-lowering medications [19, 20].

In preclinical mouse studies, we discovered that CYP46A1 could be activated by low-dose (0.1 mg/day/kg of body weight) EFV, while higher drug doses (> 0.22 mg/day/kg of body weight) inhibited CYP46A1 [21]. Low-dose EFV improved cognitive performance in 5XFAD mice, a model of AD with transgenic expression of human mutant amyloid precursor protein and human mutant presenilin 1 [22], and affected brain Aβ load. The latter effect was treatment-specific and dependent on animal age and the presence of Aβ plaques at the initiation of treatment [23, 24]. Other treatment-specific EFV effects included changes in astrocyte and microglia activation, and expression of essential synaptic proteins [24, 25]. Mechanistically, CYP46A1 activation by EFV increased the rates of the mevalonate pathway as well as sterol flux through the plasma membranes and thereby altered physico-chemical properties of plasma membranes and membrane-dependent events, such as synaptic transmission and phosphorylation of cytoskeletal and other proteins [26, 27]. In addition, increased sterol flux was shown to increase in 5XFAD mice total brain acetyl-CoA content, energetic state of brain mitochondria, and brain acetylcholine levels [27, 28]. A model of how one enzyme can control multiple and apparently unrelated processes in the brain was proposed [29, 30]. Remarkably, low-dose EFV also diminished the frequency of retinal vascular lesions that 5XFAD mice had, thus supporting further drug investigation in subjects with neovascular age-related macular degeneration [31].

The 0.1 mg/day/kg of body weight EFV dose activating CYP46A1 in mice approximately compares to a daily 7-mg EFV dose, assuming a 70-kg human. Yet, this dose cannot be used in a clinical trial without a prior pilot study as dose translation from animal to human studies is debatable [32]. Also, extant data indicate that the rates of brain cholesterol synthesis and metabolism are much higher in mice than in humans. As a result, bulk cholesterol turnover in the brain has been estimated at 0.7 years or 0.4% per day in mice [8] vs ~ 9.1 years or 0.03% per day in humans [33]. In addition, in metabolically active brain neurons of mice, cholesterol turnover was suggested to be very high, namely 20–30% per day [33, 34]. Accordingly, we hypothesized that commercially available low EFV doses (50 mg or 200 mg per day as compared to 600 mg per day approved for human use to keep HIV load low) could be used to safely activate CYP46A1 in human subjects. Furthermore, we reasoned that 20 weeks as the treatment time should be sufficient to observe CYP46A1 engagement, if human brain neurons metabolize cholesterol as actively as mouse brain neurons. To test our hypothesis, we conducted a 20-week proof-of-concept clinical research study of 50- and 200-mg daily EFV doses in patients with AD (efavirenz for patients with Alzheimer’s disease or EPAD, ClinicalTrials.gov: NCT03706885).

## Methods

### Study design

This was an exploratory randomized, double-blind placebo-controlled study with primary objectives to (1) ascertain if 50 mg and 200 mg daily EFV doses engage CYP46A1 to affect human brain cholesterol metabolism as reflected by changes in plasma 24HC levels by at least 30% (an arbitrary cutoff) and (2) confirm the safety and tolerability of 50 mg and 200 mg daily doses of EFV in older adults with cognitive impairment. A secondary objective was to precisely measure the effect of EFV on CYP46A1 activity and CNS-cholesterol turnover via stable isotope labeling kinetics (SILK). The tertiary objective was to investigate through posthoc analysis whether APOE isoforms and single nucleotide polymorphisms (SNP) in *CYP46A1* and *CYP2B6* (the latter encodes an enzyme that metabolizes EFV) affect study participant response to low-dose EFV.

### Participant recruitment and randomization

The study was conducted at two clinical sites: (1) the University Hospitals Cleveland Medical Center’s (UHCMC) Brain Health and Memory Center and the Memory Disorders Clinic in Cleveland, OH, and (2) the Alzheimer’s Clinical & Translational Research Unit at Massachusetts General Hospital (MGH) in Boston, MA.

Principal inclusion criteria for patient recruitment were as follows: age of 55–85, male or female, and diagnosed with MCI or early dementia due to AD as defined by a history of complaint of cognitive decline, a Mini-Mental State Exam score between 16 and 30 (Table 1), and a Clinical Dementia Rating global score between 0.5 and 1 [35, 36]. Principal exclusion criteria included CNS disease other than suspected prodromal or early AD, other systemic abnormalities or medications that might contribute to cognitive dysfunction, and other medications that are known to significantly alter cholesterol synthesis or metabolism in the brain, including brain penetrant statins. Detailed inclusion and exclusion criteria are presented in Supplemental Methods.

**Table 1:** Demographic and other characteristics of study participants

Characteristic	Treatment arm				
	**Placebo**	**50 mg EFV**		**200 mg EFV**	
**Patient ID**	**502–204**	**502–201**	**502-207**^a^	**501–110**	**502–202**
Age (years)	71	79	86	71	86
Gender	M	M	F	F	M
Race	W	W	W	W	B
MMSE score before treatment	27	25	26	22	16
APOE isoform status	ε3ε4	ε3ε4	ε3ε3	ε3ε4	ε4ε4
*CYP46A1* rs754203 (intron-2 T > C)	+ / −	− / −	− / −	− / −	− / −
*CYP2B6* rs3745274 (516G > T)	− / −	+ / −	− / −	+ / +	− / −
Statin use	Atorv, 10 mg/day	Atorv, 40 mg/day	No	No	No

*M* Male, *F* Female, *W* White, *B* Black, *MMSE* Mini-Mental State Exam, + present, − absent, *Atorv* Atorvastatin

^a^withdrew from the study after week 8

Randomization was stratified by clinical site, age (less than 70 years old vs 70 or greater), and gender. Double blinding was ensured by over-encapsulation of the commercially available 50 mg and 200 mg capsules of Sustiva (Bristol Myers Squibb) and by manufacturing placebo capsules by a compounding pharmacy (Lee Silsby Pharmacy in Cleveland, OH).

### Study measures

A complete list of evaluations conducted at screening, baseline, and study visits is summarized in Supplemental Table 1. All evaluations were the same at both clinical sites, except the participant at MGH underwent an additional SILK study using the protocol of deuterated water (D_2_O) administration [37] that we adapted to measure deuterium water incorporation in newly synthesized and metabolized cholesterol. This participant took 70 ml of 70% deuterated D_2_O (Sigma-Aldrich) by mouth three times a day (with at least a 3-h interval) for 7 consecutive days. D_2_O was administered twice: the first time during the week after the baseline visit and the second time during week 16 of the study. Fasting blood was drawn on days 7, 9, 14, and 21 after initiation of the D_2_O administration, as well as at weeks 4, 8, 12, 16, 20, and 22 during in-clinic visits. Plasma was obtained from the blood and used for measurements of deuterium (^2^H) incorporation into 24HC or ^2^H 24HC enrichment. Plasma processing was as described below for unlabeled 24HC, except the deuterated internal standard was omitted. Deuterated forms of 24HC were monitored by gas chromatography-mass spectrometry (GC–MS) in the SIM (selected ion monitoring) mode by peaks at *m/z* 504, 505, and 506 for one, two, and three deuterium atom incorporation into 24HC, respectively. Unlabeled 24HC was monitored by the peak at *m/z* 503, which represented the sterol molecular ion. The 24HC ion peaks at *m*/*z* 503 → 506 were then corrected for the background natural abundance of the 24HC mass isotopomers determined from the plasma samples of subjects not given D_2_O. Total ^2^H incorporation per 24HC molecule was then calculated as a percentage from the sum of unlabeled and deuterated forms of 24HC as described [38]. To normalize deuterium labeling of plasma 24HC, ^2^H incorporation into body water was measured as described [39] by plasma isotopic exchange with acetone.

Unlabeled cholesterol and 24HC were quantified by isotope-dilution GC–MS [40, 41] using a mixture of deuterated [25,26,26,26,27,27,27-^2^H_7_] cholesterol and deuterated [25,26,26,26,27,27,27-^2^H_7_]24HC that served as internal standards. Sample processing was as described [40] with the sample volumes and amounts of internal standards being 0.01 ml and 25 nmol of deuterated cholesterol for measurement of total plasma cholesterol, 0.5 ml and 0.1 nmol of deuterated 24HC for measurement of total plasma 24HC, and 1 ml for total sterol measurements in CSF, which required 2.5 nmol of deuterated cholesterol and 0.001 nmol of deuterated 24HC. Samples were mixed with 3 ml of 1.0 N KOH in 70% aqueous ethanol and saponified at 37 °C for 2 h. Then, 10 drops of concentrated HCl, 5 ml of chloroform, and 1.5 ml of 0.9% NaCl in water were added, and the mixture was vortexed and centrifuged at 1000 g for 5 min. The lower organic phase was transferred to a new glass tube, and the chloroform extraction was repeated. The organic phases from the two extractions were combined and dried in a SpeedVac. For cholesterol quantification in plasma, dried lipid extracts were trimethylsilated with 0.2 ml of bis-(trimethylsilyl) trifluoroacetamide/trimethylchlorosilane at 60 °C for 10 min and analyzed by GC–MS as described [40]. For cholesterol measurements in CSF, as well as 24HC measurements in plasma and CSF, dried lipid extracts were dissolved in 0.3 ml of methanol and loaded onto a Varian C18 column (1000 mg; Varian Inc., Lake Forest, CA) equilibrated with 5 ml CH_3_OH/CH_3_CN/H_2_O (40:40:20, vol/vol/vol). The columns were washed with 20 ml CH_3_OH/CH_3_CN/H_2_O (82:12:6, vol/vol/vol), resulting in the elution of the oxysterol fraction containing 24HC. Cholesterol was eluted by subsequently washing the column with 20 ml of methanol. All eluates were evaporated to dryness followed by trimethylsilation as described above for analysis by GC–MS. The following ions (*m/z*) were monitored in the SIM mode: 368 (cholesterol), 375 (deuterated cholesterol), 145 (24HC), and 152 (deuterated 24HC). For quantification, calibration curves were generated using a fixed concentration of the internal standard and varying concentrations of the unlabeled sterol. Each sample was analyzed once after three randomly selected samples were used to determine the coefficient of variation (CV) between three independent sample processing events. The CVs were 0.4% for plasma cholesterol, 4.5% for plasma 24HC, 4.4% for CSF cholesterol, and 2.2% for CSF 24HC.

CSF Aβ 42 and 40 peptides (Aβ_42_ and Aβ_40_, respectively), total tau, and phosphor-tau^181^ were measured by the Biomarker Core at the Massachusetts Alzheimer’s Disease Research Center using commercial ELISA kits (EUROIMMUN, Lübeck, Germany), according to manufacturer instructions. The short-term inter-visit CVs for these AD markers were normally up to 5%.

Genotyping for the APOE isoform status (ε2, ε4, or ε4) and single nucleotide polymorphisms in *CYP46A1* (rs754203) and *CYP2B6* (rs3745274) was conducted by the Molecular Biology and Genotyping Core of the Visual Sciences Research Center at Case Western Reserve University (Cleveland, OH).

### Statistical analysis

A longitudinal mixed model was used to ascertain the statistical significance of target (CYP46A1) engagement*.* Baseline and 4-, 8-, 12-, 16-, and 20-week plasma 24HC levels were included in the analysis of the four subjects who received EFV. For one subject, only baseline and 4- and 8-week plasma 24HC levels were observed, as the subject withdrew from the study. There also was one missing 4-week measurement in another subject. These missing data were implicitly imputed in the mixed model fit. We conducted a two-sided test with a type I error of 0.05. The null hypothesis was that the slope parameter associating 24HC level with a continuous time variable (weeks) was equal to 0. We did not conduct a two-group comparison between subjects that received EFV vs placebo, as there was only one subject who completed the study in the placebo group.

## Results

### Participant characteristics

Five subjects (one at the MGH site and four at the UHCMC site, described in Table 1) were enrolled in the study (CONSORT Supplemental Fig. 1). One participant was randomized to receive a placebo, two received 50 mg of EFV per day, and two received 200 mg of EFV per day. Of the five participants, four completed the study, and one (502–207, assigned to a 50-mg/day dose) withdrew from the study after week 8 due to a diffuse maculopapular rash, a known common occurrence with EFV. The four completers had at least one APOE ε4 allele, and the participant on placebo (502–204) was also heterozygous for *CYP46A1* rs754203. This SNP has been found to be associated with AD in some but not all linkage studies [42]. Of the four study subjects who received EFV, one (502–201) was heterozygous and one (501–110) was homozygous for *CYP2B6* rs3745274, a SNP associated with impaired EFV metabolism and thus high plasma EFV levels [43].

**Fig. 1:**
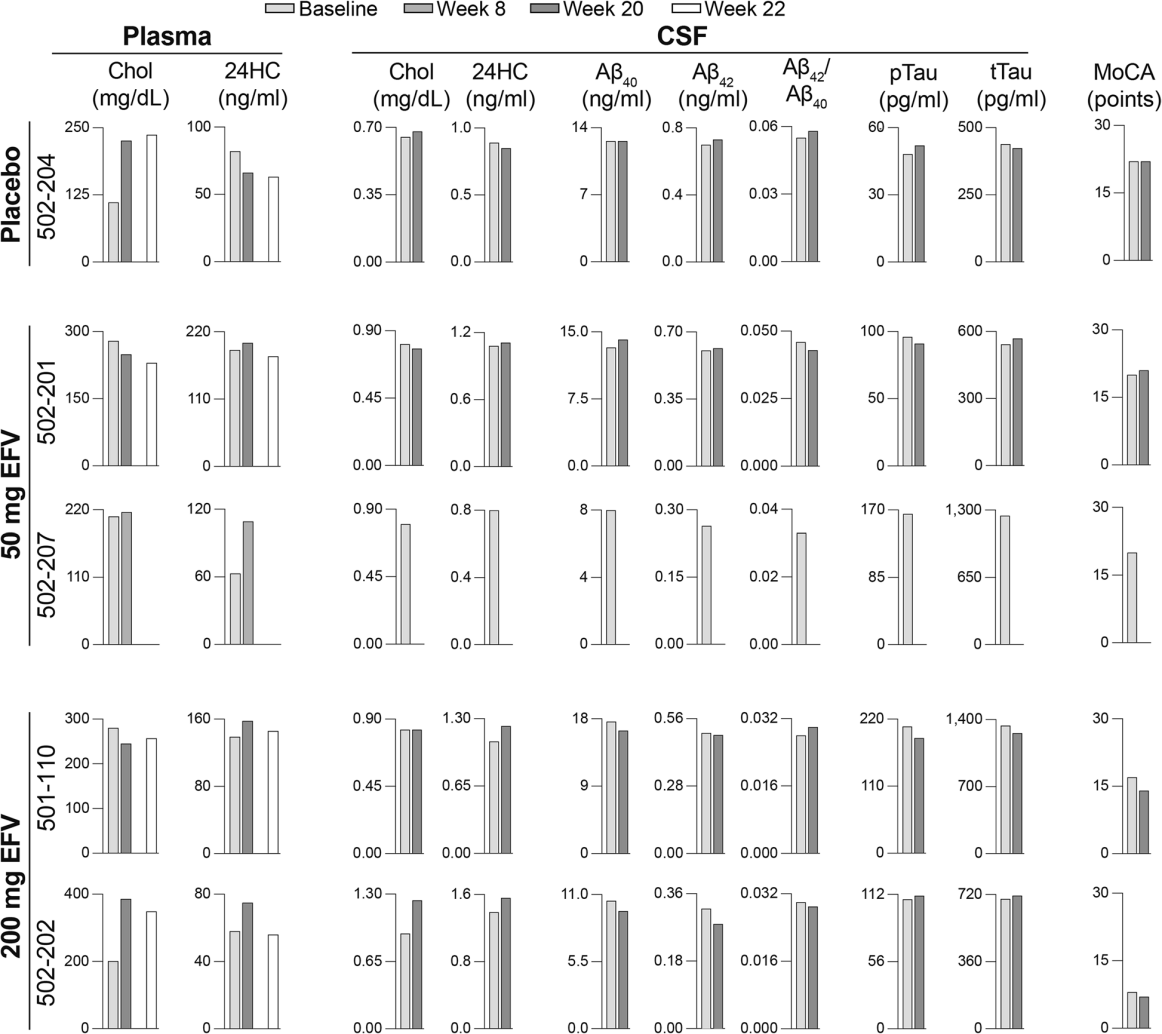
A summary of major study measures in trial participants. 24HC, 24-hydroxycholesterol; Baseline, baseline visit; Chol, cholesterol; Aβ_40_ and Aβ_42_, amyloid β peptides 40 and 42; pTau, phosphorylated tau; tTau, total tau; MoCA, the Montreal Cognitive Assessment; week 8, week 20, and week 22 in-clinic visits

### Study primary outcomes: target engagement and safety

At week 20, plasma 24HC levels were increased relative to baseline levels in the three participants on EFV who completed the study: by 6% in 502–201, 14% in 501–110, and 29% in 502–202 (Fig. 1). However, at week 22, after EFV had been discontinued for 2 weeks, plasma 24HC levels were decreased relative to week 20 in these participants, consistent with EFV wash out after its activating effect on CYP46A1 during the treatment. In addition, the participant on EFV who terminated early (502–207) also had an increase in plasma 24HC content (by 73% at week 8). The analysis of changes in plasma 24HC levels of all four participants on EFV with a linear mixed model of time (weeks) (also included participant-level random intercepts and heterogeneous variances per time period) revealed a positive association (*P* < 0.001), with slope parameter estimate of 0.828, and 95% confidence interval of (0.630, 1.025).

In the participant on placebo (502–204), who was taking 10 mg/day atorvastatin as well, serum 24HC levels were decreased at week 20 relative to baseline levels and also at week 22 relative to week 20 (Fig. 1).

Similar to the increases in the plasma, CSF 24HC levels at week 20 vs baseline were increased in the two participants on daily 200 mg EFV, by 14% in 501–110 and 12% in 502–202 (Fig. 1).

In terms of safety, both EFV doses were overall well tolerated by the study participants. There were no serious adverse events or other adverse events, such as neuropsychiatric symptoms known to occur with a daily 600 mg EFV dose in HIV [43, 44]. As noted above, one participant on a daily 50-mg EFV dose withdrew from the study due to diffuse rash felt to be consistent with a medication-induced reaction.

### Study secondary outcome: target engagement in SILK

A unique feature of the brain, namely almost exclusive cholesterol biosynthesis in situ and mostly enzymatic elimination via CYP46A1-catalyzed 24-hydroxylation [8, 10], served as the rationale for the SILK study, in which we investigated whether we could directly monitor CYP46A1 activity in the brain and brain cholesterol turnover. After oral consumption, D_2_O appears in the blood within 1–2 h [45, 46] and equilibrates within minutes with intracellular water across different organs [47]. We reasoned that deuterium atoms from D_2_O could then be incorporated into cerebral cholesterol during in situ biosynthesis and remain in the newly synthesized cholesterol during its subsequent conversion to 24HC and sterol diffusion to the systemic circulation. If so, plasma 24HC should be enriched with ^2^H, and the extent of this enrichment should reflect CYP46A1 activity and the rate of cerebral cholesterol turnover.

**Fig. 2:**
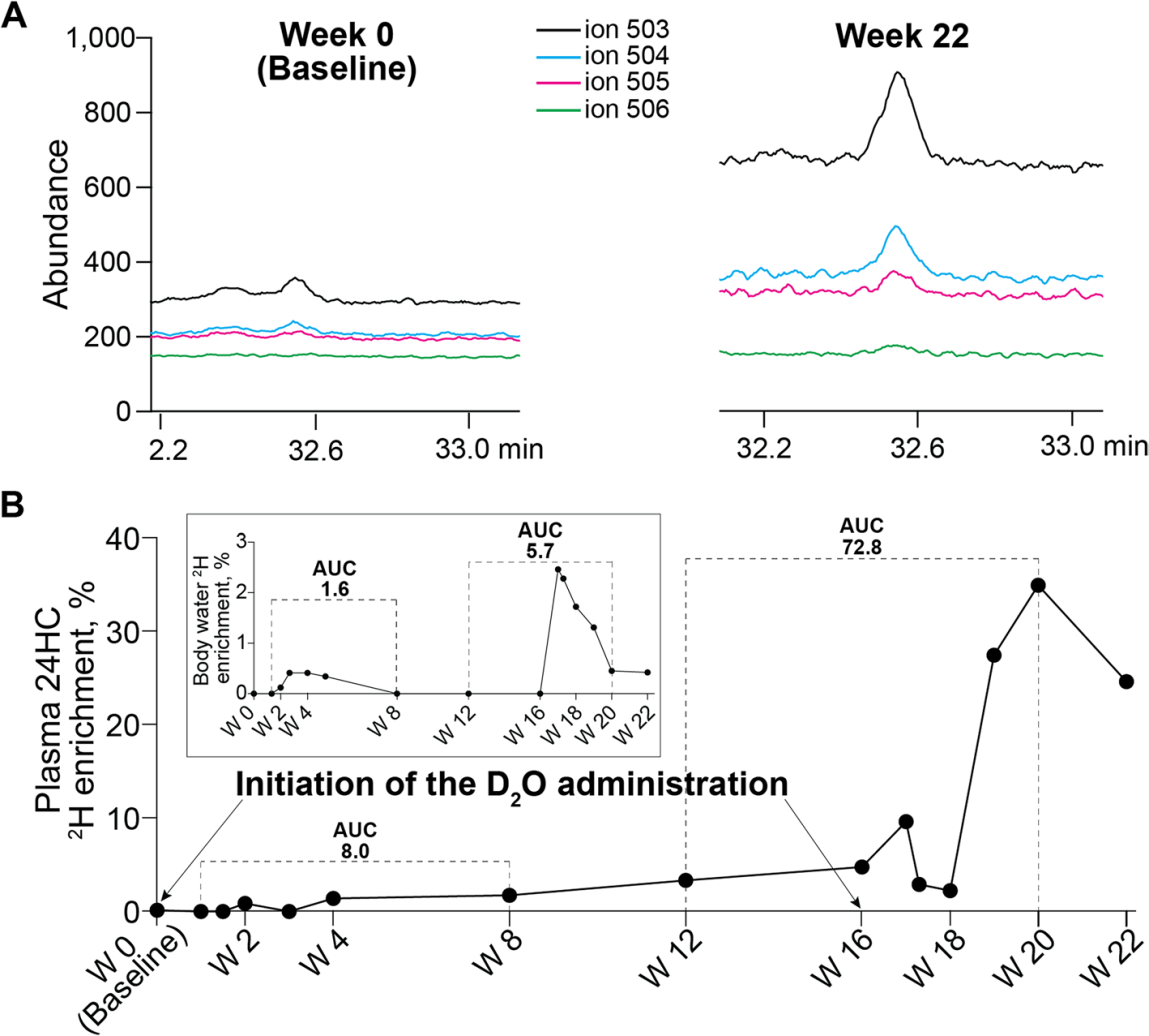
A summary of the SILK experiment. This experiment was conducted in one subject (501–110) on a daily 200-mg EFV dose. **A** Ion abundance at *m/z* corresponding to the incorporation of one (504), two (505), and three (506) ^2^H into plasma 24-hydroxycholesterol (24HC). The peak at *m/z* 503 represents the sterol molecular ion. The data for week 0 show the background natural abundance of the 24HC mass isotopomers. **B** Plasma 24HC and body water (inset) enrichment with ^2^H. The dashed line indicates the areas under the curve (AUC), which were used for the estimation of the EFV effect on CYP46A1 activity. W, week of the clinic visit. EFV treatment stopped at W 20

One participant (501–110, on a daily 200-mg EFV dose) underwent the SILK study. D_2_O ingestion by this participant led to plasma 24HC enrichment with up to three deuterium atoms (^2^H, peaks at *m/z* 504, 505, and 506, respectively, Fig. 2A) as compared to the naturally abundant mass isotopomers of 24HC measured at the baseline visit before D_2_O ingestion. Also, the ^2^H incorporation into plasma 24HC was low (up to 4.8%) but detectable during the first half of the SILK study (weeks 1–10), yet it was increased to 35% during weeks 10–20 of the study (Fig. 2B). Then, at week 22 after a 2-week washout period, total 24HC deuteration decreased by 10%, despite the fact that the deuteration extent of the body water remaining the same (Fig. 2B inset). To account for the differences in the body water ^2^H enrichment during the study, we calculated the areas under the curve (AUC) for 24HC and body water ^2^H enrichments during the first and last 8 weeks of the study. The AUC data for the plasma 24HC deuteration were then divided by the corresponding AUC data for the body water deuteration. The resulting values were 8.0/1.6 = 4.9 and 72.8/5.7 = 12.7 for the first and last 8 weeks of the study, respectively. The difference in the AUC ratios for weeks 1–8 and 12–20 was more than 2.5-fold, suggesting that EFV activated CYP46A1 in the study participant and likely also enhanced brain cholesterol turnover.

### Other study measures

These measures included changes in AD markers in CSF (Aβ_40_ and Aβ_42_ peptides; total tau and phosphor-tau^181^) as well as in the Montreal Cognitive Assessment (MoCA) scores conducted as a part of safety evaluations (Fig. 1). There were no evident changes in the measured CSF AD markers, which all were consistent with Alzheimer’s disease (the Aβ_42_/Aβ_40_ ratio < 0.0646, phosphor-tau^181^ > 42.4 pg/ml, and total tau > 342 pg/ml) nor did these correlate with plasma 24HC levels. MoCA scores did not change in one subject on placebo (502–204), but were increased by 3 points in both subjects on a daily 50 mg EFV dose (502–201) and decreased by 3 and 2 points in the subjects on a daily 200-mg EFV dose (501–110 and 502–202, respectively).

## Discussion

We investigated whether a 20-week daily treatment with low-dose EFV could activate CYP46A1 in human subjects. While being small, this study, nonetheless, suggests that EFV treatment engages and activates CYP46A1 in the human brain. First, by the end of the treatment period, participants on EFV showed a statistically significant within-group increase in plasma 24HC levels relative to baseline (uncontrolled), which then decreased after discontinuation of EFV treatment (Fig. 1). Second, the results of the SILK study were consistent with CYP46A1 activation by EFV with a 2.5-fold increase in the normalized deuterium plasma 24HC enrichment during the last 8 weeks of the study vs the first 8 weeks of the study (Fig. 2). Third, in addition to the plasma, increases in 24HC levels were observed in CSF (Fig. 1) where 24HC also diffuses from the brain [17, 18]. Collectively, these three types of measurements show that in the brains of participants with early AD, EFV activates CYP46A1 at both 50 mg/day and 200 mg/day and that a 20-week treatment is sufficient to observe this activation. Apparently, cholesterol turnover in the metabolically active brain neurons in humans is much higher than 0.03% per day, an important practical finding for future studies of CYP46A1 activation in humans. Thus, our data prompt larger clinical studies to further characterize CYP46A1 activation by EFV, including identification of the maximally effective dose for AD patients and additional insights into possible clinical efficacy in this population.

Several additional points are worth discussing. The first is a potential statin effect on plasma 24HC levels. Only 6% and 3% increases in plasma and CSF 24HC levels, respectively, were in participant 502–201, who was on a 40-mg/day dose of atorvastatin. This atorvastatin dose is known to decrease plasma 24HC levels by ~ 25% after 2 months of treatment [48]. Accordingly, it is possible that a minimal EFV effect on plasma and CSF 24HC in participant 502–201 is due to a confounding brain 24HC decrease because of atorvastatin treatment. This explanation is supported by 19% and 4% decreases in plasma and CSF 24HC levels, respectively, in participant 502–204 who was also taking atorvastatin but at a 10-mg/day dose and was on placebo. Apparently, this atorvastatin dose was not sufficient to stabilize the participant’s plasma cholesterol levels, which increased almost twice by the end of the study (Fig. 1).

Participant 501–110 was homozygous for the activity-decreasing rs3745274 SNP in *CYP2B6*, whose carriers are suggested to receive a 3-times lower EFV amount to prevent overdose [49]. This participant had a lower plasma 24HC increase relative to participant 502–202 who did not carry this SNP (Fig. 1). Thus, to have subjects with comparable EFV doses, genotyping for the rs3745274 SNP in *CYP2B6* should be included in future studies.

The administration of D_2_O to humans to trace different small molecules and proteins has been in practice since 1950 [37, 45, 46, 50]. However, we are the first group to apply this approach for the measurement of cholesterol 24-hydroxylation in the brain and CYP46A1 activation. We used two D_2_O administrations, at the beginning and end of the study, as we were not sure whether body water would still be enriched with ^2^H at the end of the study, a prerequisite for labeling of newly synthesized brain cholesterol. Indeed, body water deuteration was not detectable at week 8 of the SILK study (Fig. 2B inset). Hence, the second D_2_O administration at week 16 proved to be useful and was safe: the maximal ^2^H enrichment of body water was 2.5%, the labeling extent almost ten times lower than that (20%) considered as safe in animals [37]. The study ended at week 20, when the ^2^H enrichment of plasma 24HC was maximal (Fig. 2B). Therefore, our normalizations of the AUC for plasma 24HC deuteration to the AUC for total body ^2^H labeling represent rough estimates only. Nevertheless, they are informative and represent a more direct measure of CYP46A1 activation by EFV (and likely turnover) than plasma 24HC levels. Indeed, in a linear segment of the curve (week 4 to week 8, Fig. 2B), the average rate of plasma 24HC deuteration was 0.04% per day, which is comparable to the rate of brain cholesterol turnover (0.03% per day) determined previously in humans using a different approach [33].

The primary objective of this study was to assess EFV target engagement. Neurocognitive and neuropsychiatric assessments were not included as drug efficacy outcomes but rather were carried out for safety monitoring. No neurocognitive/psychiatric safety signal emerged.

### Limitations

A major study limitation is the low number of subjects treated with EFV, due in part, to the COVID-19 pandemic. The limited number of recruited subjects also restricted the possibility of conducting a controlled study with an adequately sized placebo comparison group. Another limitation is the enrollment of two subjects taking atorvastatin, which likely decreased the 20-week (and 22-week) plasma 24HC levels in the subject on placebo [48] and could lower the 20-week increase in the plasma 24HC content in the subject on 50 mg daily EFV. Additionally, the sample size did not allow for covariate adjustment.

## Conclusions

This pilot study of EFV (50 mg/day and 200 mg/day for 20 weeks) in subjects with early AD demonstrates proof-of-concept activation of CYP46A1, which controls cholesterol metabolism and turnover in the brain. A unique SILK protocol using deuterated water was developed to directly measure CYP46A1 activity in the brain. Collectively, the data support further studies of low-dose EFV as a potential therapeutic approach for AD.

## Supplementary Information

**Additional file 1:** Supplemental methods. **Supplemental Table 1**. Summary of visits and evaluations common for both clinical sites and specific for the MGH site. SC, screening visit; BL, baseline visit; PC, phone call; CV, clinic visit; DC, early discontinuation visit; W, week; *, MGH site only; ** unscheduled visit; 24HC, 24-hydroxycholesterol. **Supplemental Fig. 1.** A tree showing the screening, enrollment, randomization, and outcome of subjects in the efavirenz clinical trial. UHCMC, Brain Health and Memory Center and the Memory Disorders Clinic in Cleveland, OH; MGH, Alzheimer’s Clinical & Translational Research Unit at Massachusetts General Hospital.

## Data Availability

All data generated or analyzed during this study are included in this published article [and its supplementary information files].

## Abbreviations

24HC: 24-Hydroxycholesterol
Aβ: Amyloid β
AD: Alzheimer’s disease
AUC: Area under the curve
CSF: Cerebrospinal fluid
CV: Coefficient of variation
EFV: Efavirenz
GC-MS: Gas chromatography-mass spectrometry
D_2_O: Deuterated water
HIV: Human immunodeficiency virus
MGH: Massachusetts General Hospital
MCI: Mild cognitive impairment
SILK: Stable isotope labeling kinetics
SNP: Single nucleotide polymorphism
UHCMC: The University Hospitals Cleveland Medical Center
