# Supplementary material for: CYP46A1 activation by low-dose efavirenz enhances brain cholesterol metabolism in subjects with early Alzheimer’s disease
Source: Alzheimers Res Ther. 2022 Dec 29;14:198. doi: 10.1186/s13195-022-01151-z (PMC9797897; doi:10.1186/s13195-022-01151-z)
Supplement: Supplementary file 1 — Additional file 1: Supplemental methods. Supplemental Table 1. Summary of visits and evaluations common for both clinical sites and specific for the MGH site. SC, screening visit; BL, baseline visit; PC, phone call; CV, clinic visit; DC, early discontinuation visit; W, week; *, MGH site only; ** unscheduled visit; 24HC, 24-hydroxycholesterol. Supplemental Fig. 1. A tree showing the screening, enrollment, randomization, and outcome of subjects in the efavirenz clinical trial. UHCMC, Brain Health and Memory Center and the Memory Disorders Clinic in Cleveland, OH; MGH, Alzheimer’s Clinical & Translational Research Unit at Massachusetts General Hospital. [file 13195_2022_1151_MOESM1_ESM.pdf]

# CYP46A1 activation by low-dose efavirenz enhances brain cholesterol metabolism in subjects with early Alzheimer's disease

Alan J. Lerner[1,2], Steven E. Arnold[3], Erin Maxfield[4], Aaron Koenig[3], Maria E. Toth[1], Brooke Fortin[3], Natalia Mast[4], Bianca A. Trombetta[3], John Denker[4], Andrew A. Pieper[5-8], Curtis Tatsuoka[9], Sangeetha Raghupathy[4], and Irina A. Pikuleva[4]*

[1]Brain Health and Memory Center, Neurological Institute, University Hospitals Cleveland Medical Center, Cleveland, OH 44122, USA

[2]Department of Neurology, Case Western Reserve University, Cleveland, OH 44106, USA

[3]Alzheimer's Clinical and Translational Research Unit, Massachusetts General Hospital, Charlestown, MA 02129

[4]Department of Ophthalmology and Visual Sciences, Case Western Reserve University, Cleveland, OH 44106, USA

[5]Harrington Discovery Institute, University Hospitals Cleveland Medical Center, Cleveland, OH 44106, USA

[6]Department of Psychiatry, Case Western Reserve University, Cleveland, OH 44106, USA

[7]Geriatric Psychiatry, GRECC, Louis Stokes Cleveland VA Medical Center; Cleveland, OH 44106, USA

[8]Institute for Transformative Molecular Medicine, School of Medicine, Case Western Reserve University, Cleveland, OH 44106, USA

[9]Department of Population and Quantitative Health Sciences, Case Western Reserve University, Cleveland, OH 44106, USA

## SUPPLEMENTAL METHODS

*Inclusion and Exclusion Criteria*

Inclusion criteria for patient recruitment were as follows: age of 55-85; male or female; diagnosed with Mild Cognitive Impairment (MCI) or early dementia due to AD as defined by complaint of cognitive decline, the Mini-Mental State Exam (MMSE) score between 16 and 30, and the Cognitive Dementia Rating (CDR) between 0.5-1 [1, 2]. Other inclusion criteria were: education of more than 8 years; literate in English, and/or good working history that precluded consideration of mental retardation; visual and auditory acuity sufficient for neuropsychological testing; modified Hachinski Ischemic Score of less than 4 [3]; no major health issues or diseases expected to interfere with the study; willingness to complete all assessments and study procedures; not being pregnant, lactating or of child-bearing potential (women had to be >2 years post- menopausal or surgically sterile); if cognitively impaired, then have a study partner in frequent contact with the subject and willing to accompany them to visits and complete partner study forms; no contraindication or hypersensitivity to EFV; screening laboratory testing must be within normal limits or, if abnormal, must be judged to be clinically insignificant by the investigators; and use of a cholinesterase inhibitor and statin medication (except simvastatin, which seems to cross the blood-brain barrier and thus could inhibit brain cholesterol biosynthesis), if doses were stable for 3 months prior to enrollment.

The study exclusion criteria were: any CNS disease other than suspected prodromal or early AD, such as clinical stroke, brain tumor, normal pressure hydrocephalus, brain tumor, multiple sclerosis, significant head trauma with persistent neurological or cognitive deficits or complaints, Parkinson’s disease, frontotemporal dementia or other neurodegenerative diseases; ongoing major and active psychiatric disorder and/or other concurrent medical condition that, in the opinion of

the investigator, might compromise safety and/or compliance with study requirements; current suicidal ideation or history of suicide attempt; history of alcohol or other substance abuse or dependence within the past two years; any significant systemic illness or unstable medical condition that could affect study compliance, including a history of prolonged QTc; laboratory abnormalities in $B_{12}$, TSH, or other common laboratory parameters that might contribute to cognitive dysfunction; current use of medications with psychoactive properties (e.g., anticholinergics, antihistamines, antipsychotics, sedative hypnotics, anxiolytics) that, in the opinion of the investigator, might deleteriously affect cognition; use of other investigational agents one month prior to entry and for the duration of the study; treatment with any of the following agents/classes within the past 3 months: simvastatin; antiepileptic agents, clopidogrel, voriconazole, systemic ketoconazole, cyclosporine, St. John’s Wort.

# SUPPLEMENTAL RESULTS

**Supplemental Table 1. Summary of visits and evaluations common for both clinical sites and specific for the MGH site**. SC, screening visit; BL, baseline visit; PC, phone call; CV, clinic visit; DC, early discontinuation visit; W, week; *, MGH site only; ** unscheduled visit; 24HC, 24-hydroxycholesterol.

| Evaluations | SC | BL W 0 | PC W 2 | CV W 4 | PC W 6 | CV W 8 | PC W 10 | CV W 12 | PC W 14 | CV W 16 | PC W 18 | CV W 20 | CV W 22 | DC ** |
|---|---|---|---|---|---|---|---|---|---|---|---|---|---|---|
| **Administrative** | | | | | | | | | | | | | | |
| Eligibility Confirmation, Informed Consent, Demographics, Randomization, Participant ID Assignment | x | | | | | | | | | | | | | |
| Efavirenz Dispensing, Management and Return Log | | x | | x | | x | | x | | x | | x | | x |
| Concomitant Medications Log | x | x | x | x | x | x | x | x | x | x | x | x | x | x |
| Confirm Study Drug compliance | | x | | x | | x | | x | | x | | x | | |
| Protocol Deviation Log | x | x | x | x | x | x | x | x | x | x | x | x | x | x |
| **Safety and Tolerability** | | | | | | | | | | | | | | |
| Height, Weight & Vital Signs | x | x | | x | | x | | x | | x | | x | x | x |
| Medical, Surgical, Family & Social History | x | | | | | | | | | | | | | |
| Adverse Events/Active Conditions | x | x | x | x | x | x | x | x | x | x | x | x | x | x |
| 12-Lead EKG | x | | | | | | | x | | | | | x | x |
| CDR (Clinical Dementia Rating) | x | | | | | | | | | | | x | | |
| MMSE (Mini-Mental Status Exam) | x | | | | | | | | | | | | | |
| MoCA (Montreal Cognitive Assessment) | | x | | | | | | | | | | x | | |
| Modified Hachinski Ischemia Score | x | | | | | | | | | | | | | |
| NPI-Q (Neuropsychiatric Inventory) | x | | | | | x | | | | x | | x | x | x |
| Suicidality: Baseline Suicidality Assessment | x | x | | | | | | | | | | | | |
| Suicidality: Since Last Suicidality Assessment | | x | | x | | x | | x | | x | | x | x | x |
| Neurological & Physical Examination | x | | | | | x | | | | | | x | x | x |
| Symptom Checklist | | x | x | x | x | x | x | x | x | x | x | x | x | x |
| Safety Labs: Fasting CMP (Ca, BUN, Cr, AST, ALT, Bilirubin) | x | | | | | x | | x | | x | | x | x | x |
| Safety Labs: Fasting CBS with differential & Lipid Panel | x | | | x | | x | | x | | x | | x | x | x |
| Safety Labs: B12 & TSH | x | | | | | | | | | | | | | |
| Safety Labs: Urinalysis | x | | | | | | | | | | | | x | x |
| Safety Labs: PT-INR (Prothrombin Time & International Normalized Ratio | | x | | | | | | | | | | x | | |
| Lumbar Puncture | | x | | | | | | | | | | x | | |
| Urinalysis | x | | | | | | | | | | | | x | x |
| Initial Serious Adverse Events Report | | | x | x | x | x | x | x | x | x | x | x | x | x |
| Follow-Up Serious Adverse Events Report | | | x | x | x | x | x | x | x | x | x | x | x | x |
| Deuterated Water Dispensing for Seven Days* | | x | | | | | | | | x | | | | |
| **Outcomes** | | | | | | | | | | | | | | |
| Plasma: Total Cholesterol & 24HC | | x | | x | | x | | x | | x | | x | x | |
| CSF: Total Cholesterol, 24HC, Aβ40, Aβ42, Total Tau, Phosphotau | | x | | | | | | | | | | x | | |
| SILK Study Plasma: deuterated 24HC* | | x | | x | | x | | x | | x | | x | x | |
| SILK Study CSF: deuterated 24HC* | | x | | | | | | | | | | x | | |
| DNA Genotyping | | x | | | | | | | | | | | | |

CONSORT
TRANSPARENT REPORTING of TRIALS

## CONSORT 2010 Flow Diagram

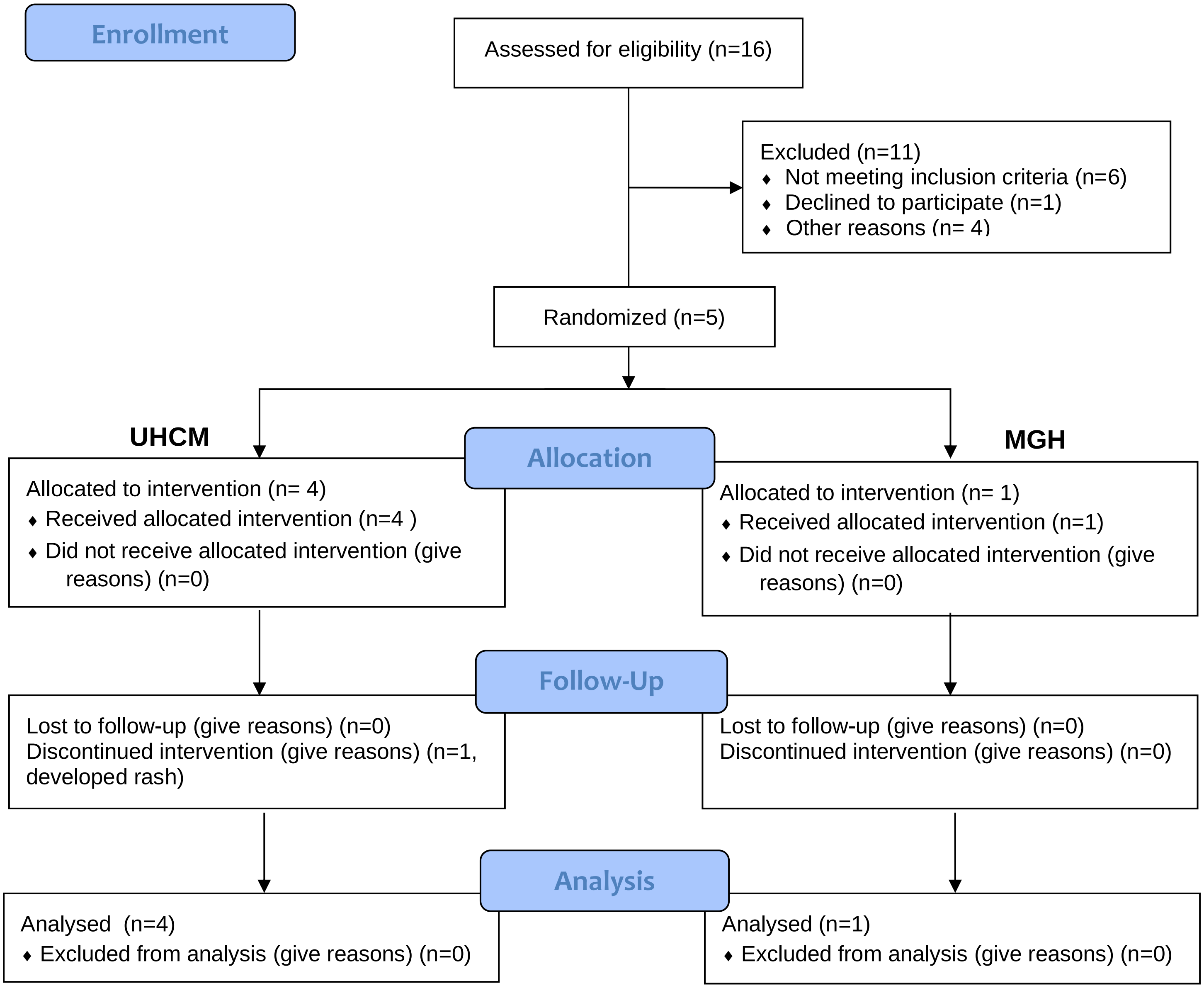


**Supplemental Fig. 1.** A tree showing the screening, enrollment, randomization, and outcome of subjects in the efavirenz clinical trial. UHCMC, Brain Health and Memory Center and the Memory Disorders Clinic in Cleveland, OH; MGH, Alzheimer's Clinical & Translational Research Unit at Massachusetts General Hospital.

**RFERENCES**


1. Folstein MF, Folstein SE, McHugh PR. "Mini-mental state". A practical method for grading the cognitive state of patients for the clinician. J Psychiatr Res. 1975;12(3):189-98.
2. Hughes CP, Berg L, Danziger WL, Coben LA, Martin RL. A new clinical scale for the staging of dementia. Br J Psychiatry. 1982;140:566-72.
3. Hachinski V, Oveisgharan S, Romney AK, Shankle WR. Optimizing the Hachinski Ischemic Scale. Arch Neurol. 2012;69(2):169-75.
